# Treatment of multiple intracranial metastases in radiation oncology: a contemporary review of available technologies

**DOI:** 10.1259/bjro.20210035

**Published:** 2021-08-31

**Authors:** Christina Skourou, Darina Hickey, Luke Rock, Peter Houston, Philippa Sturt, Siobhra O' Sullivan, Clare Faul, Ian Paddick

**Affiliations:** ^1^ St. Luke’s Radiation Oncology Network, Dublin, Ireland; ^2^ Beacon Hospital, Dublin, Ireland; ^3^ Beatson West of Scotland Cancer Centre, Glasgow, UK; ^4^ The Royal Marsden NHS Foundation Trust, London, UK; ^5^ St. Luke’s Institute of Cancer Research, Dublin, Ireland; ^6^ St. Luke’s Radiation Oncology Network, Dublin, Ireland; ^7^ Queen Square Radiosurgery Centre, National Hospital for Neurology and Neurosurgery, London, UK

## Abstract

The use of stereotactic radiosurgery to treat multiple intracranial metastases, frequently concurrently, has become increasingly common. The ability to accurately and safely deliver stereotactic radiosurgery treatment to multiple intracranial metastases (MIM) relies heavily on the technology available for targeting, planning, and delivering the dose. A number of platforms are currently marketed for such applications, each with intrinsic capabilities and limitations. These can be broadly categorised as cobalt-based, linac-based, and robotic. This review describes the most common representative technologies for each type along with their advantages and current limitations as they pertain to the treatment of multiple intracranial metastases. Each technology was used to plan five clinical cases selected to represent the clinical breadth of multiple metastases cases. The reviewers discuss the different strengths and limitations attributed to each technology in the case of MIM as well as the impact of disease-specific characteristics (such as total number of intracranial metastases, their size and relative proximity) on plan and treatment quality.

## Introduction

The incidence of brain metastases has risen over time due to the increased use of high quality neuroimaging and improvements in extracranial disease control.^1,2^ Historically, diagnosis of brain metastases was associated with poor outcomes, with treatment limited to surgery and/or whole brain radiotherapy (WBRT) or best supportive care. In recent decades however, technological advancements in radiotherapy along with greater understanding of the molecular and immunologic drivers of malignancy have resulted in a greater number of therapeutic options for brain metastases.^3–5^ Surgery and radiotherapy (RT) are often prescribed as a result of limited success of medical therapy due to the blood-brain barrier. In the presence of multiple intracranial metastases studies indicate that for certain pathologies^6,7^ stereotactic radiosurgery (SRS) targeted to each metastasis results in longer survival and reduced neurological toxicity^8,9^^,^ while for some patients there is still a role for WBRT.

The success of SRS is reliant on the sparing of healthy brain tissue from the toxic levels of radiation which are focused on the metastasis. This is often achieved by the non-coplanar delivery of multiple beams or arcs using specialised technologies to both design and deliver the treatment. SRS has found application in the treatment of functional neurological conditions (such as trigeminal neuralgia), arteriovenous malformations in the brain, and an array of benign and malignant craniospinal growths. Reports of radiotherapy for intracranial metastases date back as far as 1961.^10^ The first SRS treatment of multiple intracranial metastases (MIM) however, is reported in 1993, where “radiosurgery was undertaken repeatedly (up to five times in one individual)” to treat 160 patients with 235 cerebral metastases using a Gamma Knife.^11^ Patients were prescribed 10–56 Gy in a single fraction (mean 27 Gy) achieving local control in 94% of the cases. The authors recommended high dose radiosurgery of 1–3 metastases, suggesting that a high Karnofsky index and lack of extracranial disease improves survival. By 2014, the treatment of multiple metastases, while still not supported by clinical trials, was widely reported.^3,12,13^

The availability of technology that enables the simultaneous targeting and treatment of large numbers of intracranial metastases increased over time. Dedicated systems - such as the Gamma Knife (Elekta Instruments, Stockholm) and CyberKnife (Accuray, Sunnyvale, CA), as well as Linac-based solutions with specialised treatment planning systems (TPS) for radiosurgery, are now accessible to most clinics,^14–16^ making the simultaneous treatment of MIM common practice. As the radiosurgery community has handed these new tools, clinical trials have shown a survival benefit in the SRS treatment of 1–4 metastases that would have almost automatically been referred for WBRT in the past. Nowadays, treatment decisions are individualised based on patient- and disease-specific prognostic features,^17–20^ and include surgery,^21^ SRS^22–26^ hypofractionated stereotactic radiotherapy (FSRS),^27^ WBRT in its various forms (with or without hippocampal sparing or/and simultaneous integrated boost),^28–30^ and targeted and systemic therapies.^31,32^ For patients in whom longer-term survival is expected, there has been a shift from the widespread use of upfront WBRT to one of a more localised approach with SRS alone,^33,34^ supported with evidence from randomised controlled trials for patients with limited (1–4) brain metastases and favourable prognostic features.^24–26,35,36^ For patients with more extensive (>4) brain metastases practice is also evolving.^37–39^ Retrospective and single institution prospective studies suggest that treatment with SRS is effective and safe without WBRT.^40–45^ Total tumour volume as opposed to the absolute number of metastases appears to be a more meaningful metric for prognostication, though the maximum volume and/or number best treated with SRS remains unknown. There are randomised trials currently recruiting, with some studies examining treatment of up to 20 metastases.^46–52^ Continued management with radiosurgery is also increasingly offered, where new metastases detected on follow-up surveillance imaging are radically treated as they develop, reserving WBRT only for miliary or leptomeningeal disease.

This paper presents the most commonly used platforms for planning and delivering SRS to MIM. It demonstrates each one’s characteristics using clinical cases, offering a review of current issues around the treatment of MIM such as relevance, feasibility, plan evaluation metrics, and optimisation techniques.

## Methods and materials

**Figure 1.**
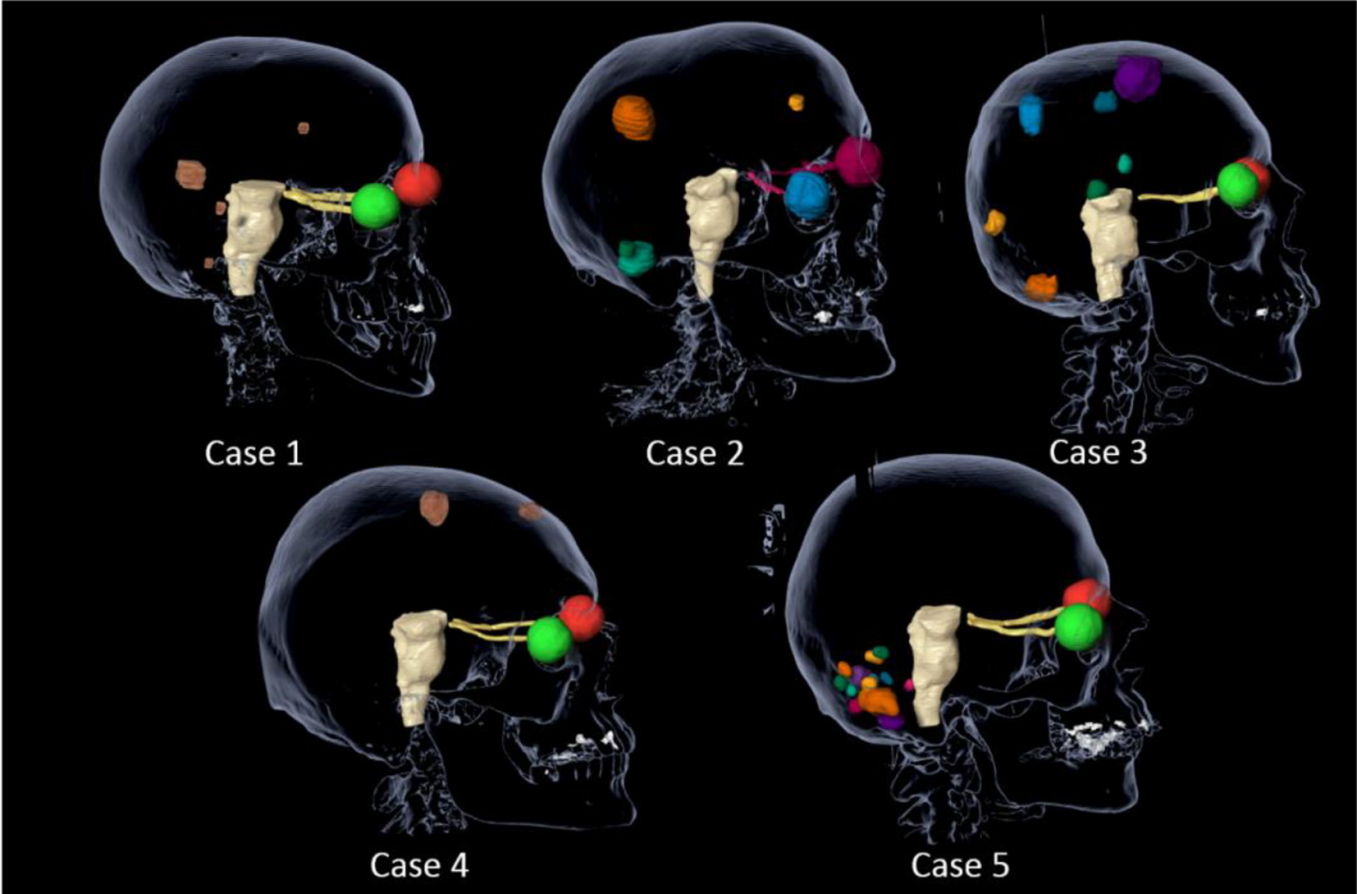
Clinical cases.

Platforms and their respective TPS were (in no particular order): Gamma Knife Icon (GammaPlan v. 11; Elekta AB, Stockholm, Sweden), linac-based systems (A. Trilogy 6SRS (HD120) with Elements Multiple Metastases v. 1.5, Brainlab, Munich, Germany; B. TrueBeam 6FFF (HD120) with Eclipse RapidArc/HyperArc v. 15.5.07, Varian Medical Systems, Palo Alto, CA; and C. Trilogy 6SRS (conical collimators) Cone Planning v. 11), and CyberKnife VSI (6FFF with Precision v. 2.0.1.1, Accuray, Sunnyvale, CA). Five clinical cases were selected to be technique agnostic and to represent a wide spectrum of scenarios as described in Table 1/Figure 1. Each case was planned by one of the authors, as expert planners, to be clinically acceptable and deliverable, using the TPS listed above as a vehicle to demonstrate each technique’s performance and attributes.

**Table 1.** Short description of representative clinical cases of multiple metastases

*Case no.*	*1*	*2*	*3*	*4*	*5*
***No of metastases***	4	4	7	2	14
***Total tumour vol[cm^3^]***	2.538	7.717	9.819	3.275	5.741
***Min tumour vol [cm^3^]***	0.077	0.146	0.453	0.964	0.034
***Max tumour vol [cm^3^]***	2.219	4.794	3.509	2.311	2.898
***Short description***	Metastases of varying volumes requiring different fractionation schemes	Distant and adjacent metastases of varying volumes	Disperse metastases with one lesion adjacent to an organ at risk	Simple case of 2 metastases	Large number of metastases confined to cerebellum

OAR, organ at risk.

The metastases were outlined by a radiation oncologist (CF) experienced in intracranial radiosurgery. For the purposes of this exercise, a 1 mm margin was applied to each metastasis even though Gamma Knife and CyberKnife plans do not usually add a margin. The prescriptions were based on target diameters as follows:<1cm: 24 Gy in 1 fraction, 1–2 cm: 20 Gy in 1 fraction;>2cm: 27 Gy in 3 fractions. Each planner strived to achieve 99% coverage of the planning target volume (PTV) while respecting the dose–volume constraints (DVC) in Table 8,^53,54^ and produce both a clinically acceptable and technically deliverable plan.

## MIM Platforms and Treatment Planning Systems utilized in this study

Five TPS, each with its own intrinsic capabilities and planning philosophy, supporting three different platforms are discussed in this review. These platforms and TPS are used as representatives of each of the categories of cobalt-based, linac-based, and robotic platforms. The following is a brief description of each technology with a focus on its application in the treatment of MIM. Table 2 lists the dedicated platforms and TPS most commonly used for the planning and delivery of intracranial SRS.

**Table 2.** Inventory of dedicated platforms and planning systems used for the treatment of multiple intracranial metastases

Treatment platform	Treatment planning system		Dose calculation algorithm	Delivery	Module within TPS
	Name	Manufacturer			
Gamma Knife	GammaPlan	Elekta	TMR/ Convolution	Cones	
Linac	Elements	Brainlab	PB/MC	Cones (arc)/ DCA	Multiple Brain Mets
	Eclipse	Varian	TMR	Cones (arc)	Cone Planning
	Eclipse	Varian	AAA/Acuros	VMAT	HyperArc
CyberKnife	Precision	Accuray	Ray-Tracing (High Resolution)	Cones/MLC non-isocentric robotic	

AAA, Anisotropic Analytical Algorithm; DCA, Dynamic Conformal Arcs; MC, Monte Carlo; PB, Pencil Beam; TMR, Tissue Maximum Ratio; VMAT, Volumetric Modulated Arc Therapy.

### a. Cobalt based platform:Gamma Knife

**Figure 2.**
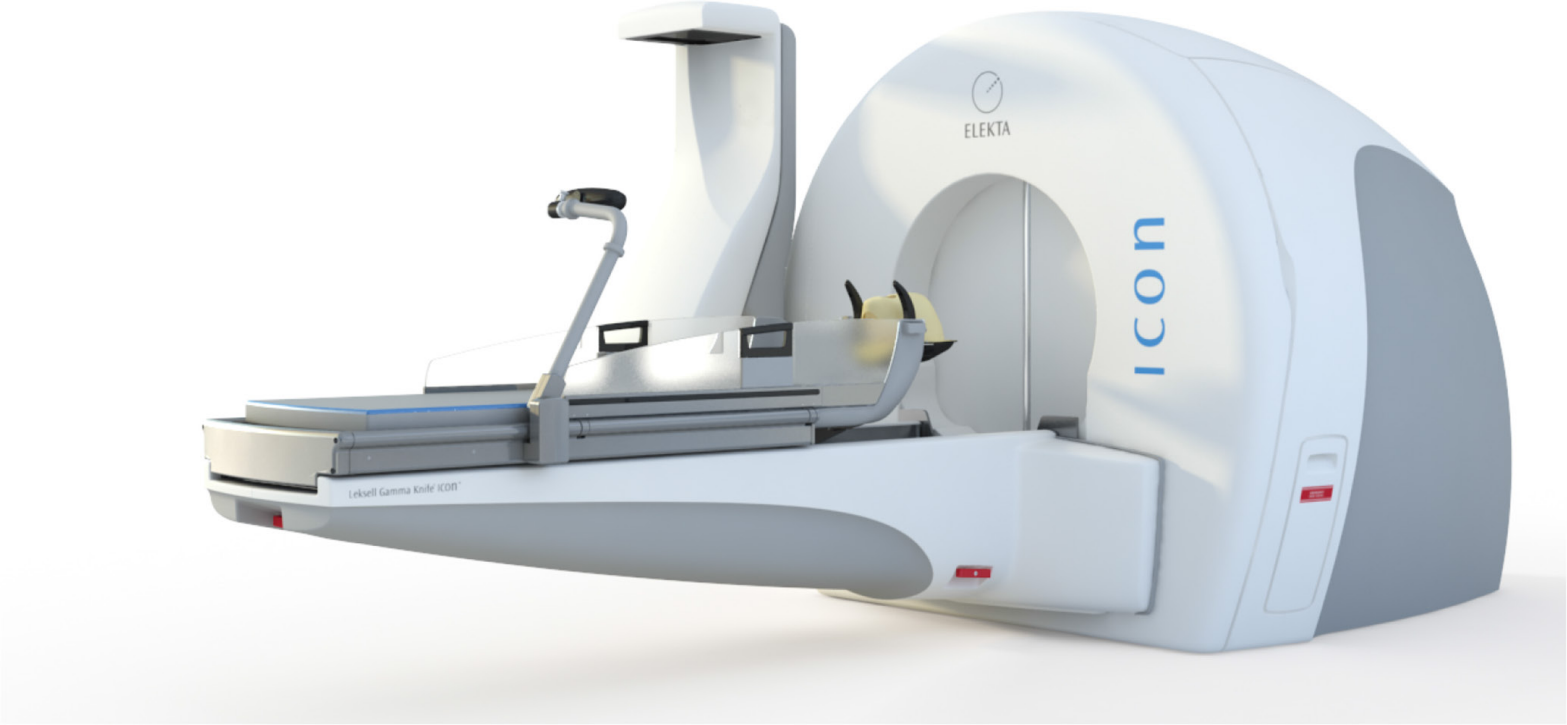
Cobalt based platform (Leksell Gamma Knife(R) Icon (TM).

In the modern day Gamma Knife Figure 2, 192 1-mm diameter Cobalt-60 sources and their corresponding collimators are arranged in a cone section configuration that approximates five non-coplanar arcs. The radiation unit is subdivided into 8 sectors of 24 sources each, which can move independently over the three different collimator apertures of 4, 8 and 16 mm.^55^

The technique of manual planning has been well described previously^54^ and requires the placing of multiple isocentres, or ‘shots’ of different diameters into the target, in order to create a prescription isodose that conforms to the size and shape of the lesion. 1–50 isocentres are typically used to cover a single target, corresponding with around 200–10,000 beams respectively. For the treatment of MIM, a single isocentre per target is often used. Conformity can be increased by combining different diameter beams into a single isocentre, which adjusts the focus to the size and shape of the lesion. For the manual planning of MIM, lesions are typically planned on an individual basis with a composite plan being calculated in the final stages of planning, taking into account the cross-talk between individual targets. However, convex optimisation inverse planning modules are now available, and are becoming an increasingly sophisticated alternative to manual planning.^56,57^

Treatment of all lesions is delivered automatically in one session. Despite the large numbers of lesions that can be treated, brain doses tend to be surprisingly low, with mean doses rarely exceeding 3 Gy.^58^ This has been partially attributed to the low prescription isodose that characterises Gamma Knife treatments (between 40 and 55% of the maximum dose), as this can increase dose gradient, reduce the beam-on time and often improve conformity.^59^

Patient immobilisation is traditionally via the Leksell Frame, secured to the outer table of the skull. Movement between isocentres is enabled by the Patient Positioning system, which has a repeatability of better than 0.05 mm,^55^ achieving a target accuracy <0.5 mm.^60^ This accuracy, combined with rigid frame fixation, is why Gamma Knife treatments are not delivered with an additional margin. Most patients have the whole process of frame fitting, imaging, treatment planning and treatment performed in a single day on an outpatient basis. While treatment for MIM can take up to several hours, this is anecdotally reported to be well tolerated. Mask-based treatment enables easy hypofractionation if required.

### b. Linac-based platforms:

**Figure 3.**
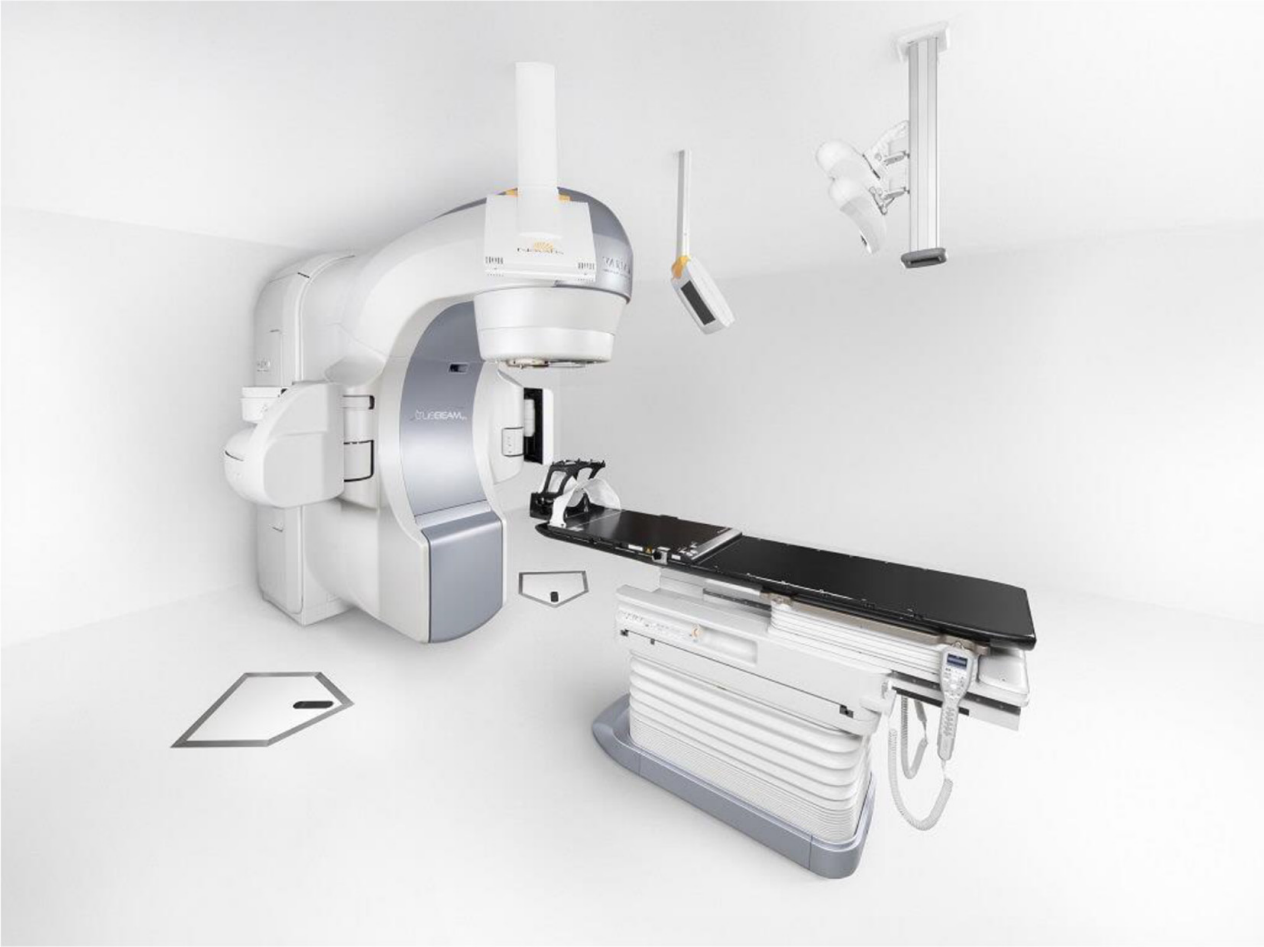
Linac based platform (Varian TrueBeam (TM) fitted with Brainlab ExacTrac(R) patient positioning and monitoring system).

Linac-based stereotactic radiosurgery Figure 3 typically uses 6 MV high dose-rate (1000–1400 MU/min) beams shaped by attached conical collimators or multileaf collimators (MLC) with the patient historically immobilised in a rigid frame.^61^ Recent technological advances, including rigid thermoplastic face mask systems, embedded microMLC (2.5–5 mm width), on board imaging, 6 degrees of freedom couches, intrafraction surface guidance or X-ray-based monitoring and positioning systems make the linear accelerator an accessible platform for SRS.^62^

#### i. Conical collimators

The simplest linac modification to allow for treatment of small lesions in the brain uses conical collimators. The availability of collimator diameters from 4 mm to over 25 mm gives this method the desirable flexibility to conform to spherical or oblong targets while achieving sharply defined field edges. The linac-based conical collimator solution (CC) commercially available by Varian Cone Planning TPS benefits from the added degrees of freedom created by couch-gantry combinations. Traditionally, linac-based SRS prescriptions are to the 80% isodose line, resulting in heterogeneous distribution inside the target with Dmax of up to or exceeding 120% of the prescribed dose increasing the dose fall off beyond the field edge, even though it was recently reported that prescriptions to lower isodose lines give a more optimal dose fall-off.^63,64^

In the case of MIM, planning and treatment delivery are limited to one lesion per isocentre or even multiple isocentres per lesion. Delivery is prolonged by the need for couch and gantry movement, resulting in lengthy treatment times, which may be multiplied by as many treatment sessions as there are intracranial metastases for a single patient. Despite this limitation, the accessibility and affordability of this linac-based solution has made SRS achievable for most community-based clinics.

#### ii. Non-coplanar DCA

The impracticality of using multiple-size conical collimators to achieve dose conformity in non-spherical targets along with the improvements in the design of MLCs lead to the development of fine-resolution MLC leaves to closely approximate the smooth edge of custom shielding blocks.^65^ Nonetheless, MLCs provide, to some extent, a slightly wider physical beam penumbra than custom shielding blocks and conical collimators^66^ and introduces additional uncertainty in the definition of the field edge potentially resulting in the need for an additional margin.

Most intracranial metastases are characterised by low geometric complexity with limited numbers of proximal organs at risk (OARs) making the dynamic conformal arc (DCA) the natural evolution of conical collimators adopted by Brainlab in their Elements Multiple Brain Metastases TPS. DCA utilises MLCs to conform to the outer contour of the target providing protection of the surrounding normal tissue while prioritising the delivery of dose to the target. While this approach may not be ideal for complex targets, the geometric simplicity of intracranial metastases makes this solution quite attractive.^67^ The low degree of MLC modulation lends itself to simpler dosimetry, faster delivery and monitor unit economy. The use of the linac’s primary and secondary collimators for SRS increases the field size and therefore target size that can be treated, allowing for the first time the simultaneous treatment of multiple targets via the same arc.

Prescription isodose and heterogeneity are comparable to those for conical collimators. The accuracy of delivering the treatment with MLC is comparable to linac-based conical collimator approaches; however the constant motion of the MLCs introduces additional uncertainty with regards to the field edge. Considering these uncertainties alongside radiation and imaging isocentre coincidence, it is therefore more common to see PTV margins in the order of 1 mm when delivered using modern linac modalities, something that was rare for cone-based solutions.

The main benefit of the MLC solution is the improvement in conformity index especially in larger lesions.^53^ For MIM, the high conformity can be maintained while simultaneously treating numerous targets, thus significantly reducing the treatment time in comparison to cone based solutions.

#### iii. Non-coplanar VMAT

Similar to DCA, the use of non-coplanar fields and carefully considered collimator angles are employed in volumetric arc therapy (VMAT) to yield conformal plans.^68,69^ Wu, Snyder et al 2015^70^ developed an algorithm which automatically optimises the couch, collimator and gantry angles to reduce normal brain dose without significantly affecting conformity index and homogeneity index. Such automation is embedded in the Varian Eclipse HyperArc TPS to ensure optimum planning geometries without compromising on efficiency. As in the case of DCA, a single isocentre can be used to treat multiple metastases; the gantry, collimator and couch positioning automation alongside MLC modulation can result in more complex plans that better spare the normal tissues and OAR proximal to the target. The automated approach can also achieve multiple prescriptions within the same treatment plan and target more lesions with a single setup.

### c. Robotic platform: CyberKnife

**Figure 4.**
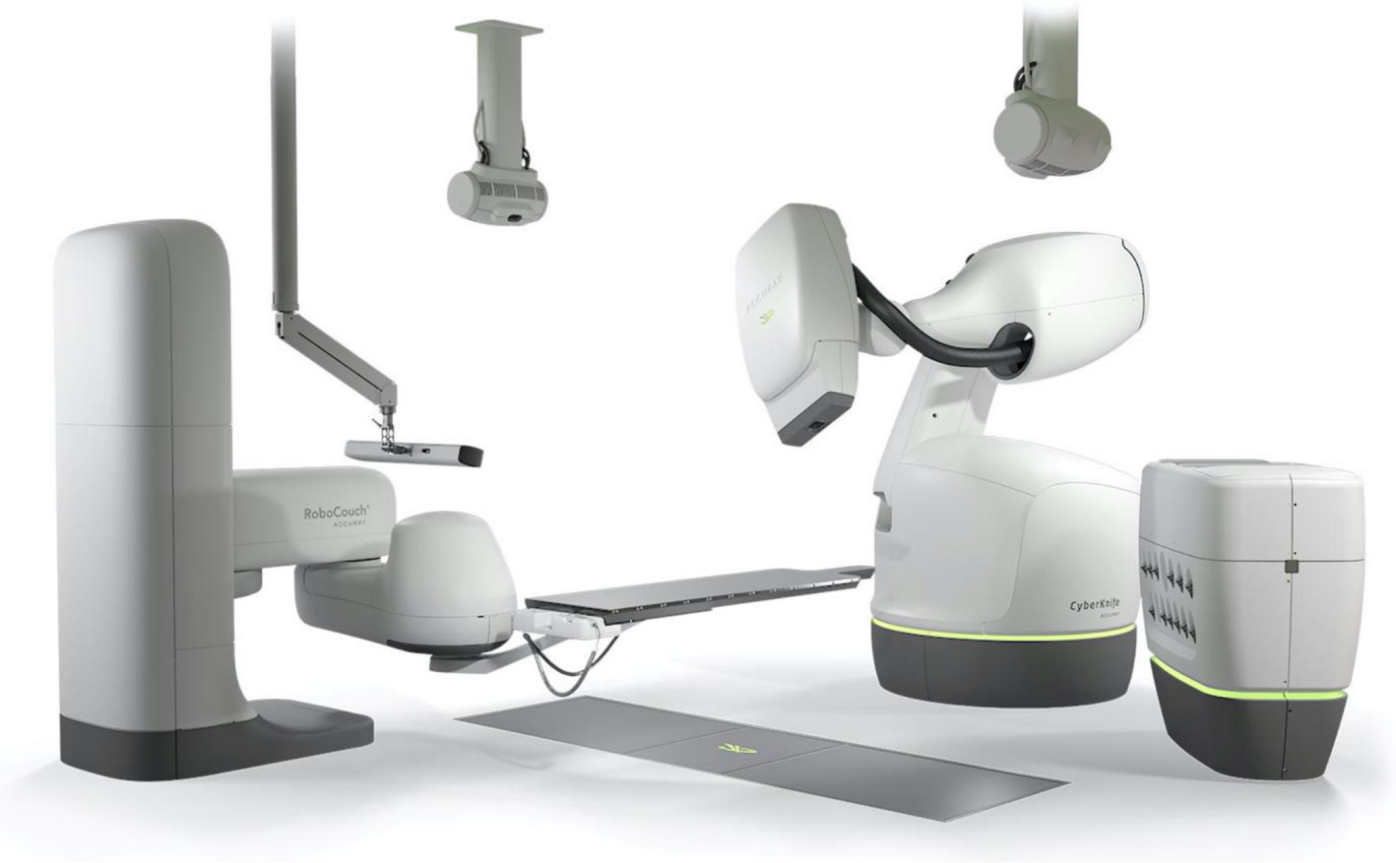
Robotic platform (Accuray CyberKnife(R) M6 System)

CyberKnife Figure 4 is specifically designed for the delivery of intra- and extracranial radiosurgery treatments. Patient immobilisation for MIM treatment is achieved using thermoplastic shells and target tracking is attained using the 6D skull feature-based tracking method. X-ray images are taken every 15–150 s, and deviations from reference images can be corrected by the robotic-arm without interrupting treatment.

Treatment planning is performed using Accuray MultiPlan or Precision with VOLO optimisation which offers the use of unlimited structures for planning, enabling the treatment of a number of metastases in a single session. Plan sums are available where multiple plans are required due to a difference in dose fractionation. Additionally, the PreciseRTX retreatment module allows deformable registration, contour transfer and dose summing between current and previous treatments. For the planning of MIM, multiple collimators can be selected for each target individually, with the planner managing the balance between conformality for small or irregular metastases, and faster treatment for larger, more spherical metastases. Planning all targets within the same plan allows improved control of low dose spread and cumulative dose to OARs relative to earlier versions of the TPS.

Treatment plans consist of typically >100 non-coplanar, non-isocentric beams collimated using either fixed cones or the variable aperture Iris collimator. For both collimation systems, aperture sizes range between 5 and 60 mm although in practice Iris is usually not selected for field sizes smaller than 10 mm. The M6 model additionally has a set of InCise^™^ MLCs with 2.5 mm leaf width. All treatment machines are 6 MV flattening filter free, with dose rates of 800–1000 MU/min. A typical single metastasis plan would take about half an hour to deliver, but treatment time increases rapidly with the number and complexity of the metastases. Treatments for larger numbers of metastases can take several hours.

## Discussion of clinical cases and clinical evaluation of MIM treatment platforms and TPS

The platforms and planning systems, listed in Table 2 have been dosimetrically compared at length in literature both in the context of treating a single lesion and more recently for MIM.^71–77^ Planning studies using a single case planned across multiple treatment planning systems have also been completed^78^ focusing differet planning techniques but excluding the impact of the technology delivering the plan. The strength of evidence demonstrating superiority in plan or treatment quality of one platform over the other has been low.

This review is mainly focused on the technologies for delivering radiosurgery to MIM. Inevitably, some technologies are paired with corresponding TPS, and therefore the two cannot be uncoupled. For the purpose of enabling discussion, and not as direct planning or dosimetric comparison between platforms, the following plan quality metrics were evaluated for the five cases described in Section 3: the RTOG Conformity Index,^79^ Gradient Index^59^ maximum dose inside the target, and the V_12Gy_ (single fraction), V_18Gy_, and V_24Gy_ (for three fractions) to normal brain tissue.^53,80,81^

Tables 3–7 quantitatively describe the results from the planning exercise. Case 5 was not included in the analysis as the proximity of the metastases to each other deemed inaccurate determination of the above parameters for each individual metastasis. It can be argued that this case is more appropriate for whole-cerebellum/posterior fossa RT. This case, was selected to demonstrate the challenge of SRS in treating lesions in close proximity as well as offer it as a solution when whole-cerebellum/posterior fossa RT is not available, *e.g.* following WBRT. All plans produced by all TPSs comfortably met the OAR dose–volume constraints set in Table 8 and adequately covered the targets with the prescribed dose (PTVRx >99%). These reported values are representative of the plans generated routinely generated by the participants at their home institutes with the exception that Gamma Knife and CyberKnife plans are usually not treated with a margin.

**Table 3.** Conformity Index: The RTOG Conformity Index (CI) is defined as: [V_PIV_/V_TV_] Where TV is the target volume and PIV is the prescription isodose volume

*CI*	*Elements*	*Cyber* *Knife*	*Eclipse CC*	*Hyperarc*	Gamma *Knife*
***AVG***	1.46	1.25	2.03	1.12	1.12
***SD***	0.29	0.43	0.38	0.14	0.04

Traditionally, a conformity index of just above 1.0 is considered optimal, as values increasingly greater than one imply greater spillage of the prescription isodose outside the target.

**Table 4.** Gradient Index: The Gradient Index (GI), is defined as: [ V_PIV50%_ /V_PIV100%_] where PIV_50%_ is the volume of the 50% of the prescription isodose and PIV_100%_ is the volume of the prescription isodose

*GI*	*Elements*	*Cyber* *Knife*	*Eclipse CC*	*Hyperarc*	Gamma *Knife*
***AVG***	3.32	3.73	2.91	3.54	2.99
***SD***	0.68	0.86	0.51	0.89	0.5

The lower the GI, the steeper the gradient. A Gradient Index of <3.0 was originally proposed as being optimal.

**Table 5.** Maximum dose inside the PTV as a percentage of the prescription dose

*Dmax (%)*	*Elements*	*Cyber* *Knife*	*Eclipse CC*	*Hyperarc*	Gamma *Knife*
***AVG***	121.9	171.5	138.0	139.2	216.0
***SD***	11.6	18.7	15.4	10.3	27.2

The maximum dose indicates the heterogeneity of dose within the target. The pros and cons of having a high dose inside the target are hotly debated, but there is inadequate clinical evidence at present to verify its importance. A lower prescription isodose will increase the dose to the centre of the target, which may be hypoxic and radioresistant, improving local control. On the other hand, a higher dose may precipitate symptomatic radionecrosis.

**Table 6.** Combined normal brain tissue volume receiving above indicated dose (Brain - GTV)

*Normal brain tissue volume [cm^3^]*	*Elements*	*Cyber* *Knife*	*Eclipse CC*	*Hyperarc*	Gamma *Knife*
***>12*** Gy ***(1 fraction)***	10.4	7.1	11.4	19.9	6.3
***>18*** Gy ***(3 fractions)***	21.4	10.5	41.7	16.5	17.6
***>24*** Gy ***(3 fractions)***	12.5	9.3	25.5	7.5	4.3

GTV, gross tumour volume.

**Table 7.** Dose to brainstem for plans created for Case 3 prescribed 27 Gy delivered over 3 fractions to the metastasis abutting the brainstem

*Brainstem dose [Gy]*	*Elements*	*Cyber* *Knife*	*Eclipse CC*	*Hyperarc*	Gamma *Knife*
***0.035 cm^3^***	26.5	22.55	30.2	27.3	28.7
***0.5 cm^3^***	19.77	17.87	22.75	17.9	16.8

**Table 8.** Dose–volume constraints used in planning of the clinical case^53,82^

***Dose–volume constraints***	*One fraction*	*Three fractions*
***Normal brain tissue [Brain - metastasis, cm^3^]***	V12Gy < 10 cm^3^	V24Gy < 10 cm^3^ V18Gy < 30 cm^3^
***Brainstem***	D0.035cm^3^ <15 Gy D0.5cm^3^ <10 Gy	D0.035cm^3^ <23.1 Gy D0.5cm^3^ <18 Gy
***Optic pathway***	D0.035cm^3^ <10 Gy D0.2cm^3^ <8 Gy	D0.035cm^3^ <17.4 Gy D0.2cm^3^ <15.3 Gy

Normal brain tissue constraints were applied to individual metastases. When the respective isodose line was encompassing more than one metastasis, the constraint was applied to the combined volume.

It is evident from the tabulated parameters that the lowest conformity index (CI) is shared by Gamma Knife and HyperArc (non-coplanar VMAT), while the fastest dose fall-off (gradient index, GI) is achieved when using conical collimators (both linac-based CC and Gamma Knife). The combination of conformity and gradient index combined represents dose spillage and traditionally sufficed to describe the quality of a plan. With the difficulty associated with deriving these indices for neighbouring lesions, however, these indices and their clinical significance must be re-examined in the context of MIM. Gamma Knife and CyberKnife create more inhomogeneous plans as they prescribe to lower isodose lines, something that in our series did not appear to impact mean GI in MIM, but may have impacted the low dose to normal tissue. The risk for necrosis with volume-dose has been documented for different fractionations in the treatment of a single intracranial lesion.^53,83,84^ However, the data for a fragmented brain volume–dose are not yet conclusive. Therefore, a comparison between plans based on combined normal tissue volume cannot be clinically argued. In the example cases, linac attached conical collimators provide a high dose gradient but increased combined normal tissue V12Gy, whereas CyberKnife produces a gradient not as steep but results in a much smaller combined normal tissue V12Gy. Furthermore, there is little evidence that a heterogenous dose distribution is either beneficial or detrimental,^85–87^ though there is some evidence to suggest that steering the dose peak inside the lesion affects the type of pathophysiological response of treatments with the same peripheral dose prescription. Additional factors previously reported in literature to affect the equality of the achieved plans in the context of MIM, emerged from the completed case studies.^88,89^ These were the number of metastases and the distance between metastases, the size of individual metastases, and their proximity to OAR such as the optic pathway or the brainstem. Note that these parameters are patient-specific rather than treatment platform- or TPS-specific.

The distance between metastases is a decisive factor in the ability to spare normal tissue in between the lesions. Case 5 is an example of MIM where normal tissue cannot be spared by dose manipulation regardless of platform used and a fractionated regime may need to be prescribed to meet our DVC. Case 2 similarly challenged all TPS with the size and proximity of the two cerebellar targets (shortest target edge-to-edge distance <5 mm). The unavoidable dose contribution from the treatment of each target would make the summation of the dose to the normal brain tissue impossible unless the same fractionation was used. To avoid this scenario, the larger metastasis determined the fractionation for both. Previous treatment delivered to the patient either in the form of WBRT, SRS, or fractionated radiotherapy must be taken into account, as this may determine the prescription regardless of the treatment technique’s abilities.^90^ All systems have the ability to import previously delivered plans, with HyperArc and Precision also sharing the advantage of being able to account for them during planning. Dose, however, of plans prescribed with different fractionations and in different treatment events cannot be simply summed and must be done with extreme caution as to not mislead the evaluation of dose to the OAR. Where targets are close together, it is usually best to keep them in the same plan (and thus prescribe the same fractionation) in order to best control the dose in the region between them; this strategy was used for Cases 2 and 5 by all planners.

In addition to the challenges posed by targets that are in close proximity, targets that are far from each other have their own issues (Cases 1 and 3). For the platforms that treat MIM with a single isocentre, poor isocentre placement can lead to compromised coverage during treatment, particularly without the use of 6 dof corrections and intrafraction monitoring systems.^67,91,92^ Gamma Knife and linac-based CC platforms are immune to this issue as the isocentre is individually placed inside each target and CyberKnife treatments are non-isocentric. For these platforms, large numbers of mets can take considerable time to deliver (CyberKnife utilises about 10 times more MU than Elements DCA to deliver the same prescription).

With linac-based systems, pre-treatment imaging, rigid immobilisation systems, and surface-guided radiotherapy (SGRT) or kV imaging systems capable of imaging at non-coplanar angles (*e.g.* Brainlab ExacTrac^TM^) can correct for intrafraction translations and rotations and minimise motion during linac-based treatment.^74^ With the latest version of Gamma Knife (Icon), CBCT guidance is available, with infrared real-time monitoring of patient position. All CyberKnife models have online orthogonal kV imaging and utilise the 6D skull feature-based tracking method to monitor patient position throughout treatment.

OARs are often easy to respect in the treatment of MIM and may be managed by adjustments in the fractionation regime. When a target is adjacent—or embedded—to an OAR as in Case 3, the balance between target coverage and OAR sparing may be more challenging for single isocentre MLC-based systems, especially as distance to isocentre can affect the accuracy of the dose delivery.

## Conclusions

Innovation and advancement in automated treatment planning software have given rise to the potential to improve the efficiency of RT planning and treatment delivery for multiple intracranial metastases. Specialised platforms such as Gamma Knife and CyberKnife continue to provide optimal solutions at the expense of treatment time, while workhorse linacs can be adapted to provide comparable results improving access and efficiency to SRS.

This review demonstrates that in the case of MIM, disease-specific characteristics (*e.g.* total number of intracranial metastases, their size and relative proximity) have more impact on plan quality than the technologies themselves. Overall, for patients with multiple brain metastases which are treatable with SRS from a technical standpoint, the actual effectiveness of SRS is primarily a function of proper patient selectionas opposed to the planning and delivery system use, taking into account not only the intracranial disease but also performance status, extracranial disease burden and control, and overall prognosis given the molecular drivers of the disease and systemic treatment options available. Multiple brain metastases are a common clinical scenario, though the management is increasingly complex. Where the optimal management includes the delivery of radiosurgery, in 2021 the technology is available and fit for purpose.
